# Managing charity 4.0 with Blockchain: a case study at the time of Covid-19

**DOI:** 10.1007/s12208-021-00281-8

**Published:** 2021-03-03

**Authors:** Adalberto Rangone, Luca Busolli

**Affiliations:** 1 University G. D’Annunzio of Chieti-Pescara, Viale Pindaro, 42, 65127 Pescara, Italy; 2 Genova, Italy

**Keywords:** Innovation, Blockchain, Fundraising, Digital transactions, Development strategies

## Abstract

The Covid-19 emergency is demonstrating the need to follow new solutions that can support the important role played by non-profit organizations around the world. Contrary to what should have happened to further combat the effect of pandemic, the majority of philanthropic organisations had a negative impact on fundraising, suffering a substantial decrease. Today, the Blockchain can play a pivotal role to re-establish pre-pandemic standards and enhance the development of global philanthropy. However, it is still too little considered due to the criticalities encountered during the launch and development of the initiatives as well as for a general incomprehension of its technology. Therefore, this work aims to demonstrate the Blockchain impact on the development of charity 4.0, especially in an extremely dramatic historical moment marked by the Covid-19 pandemic. The objective is achieved through the case study of Charity Wall, an emerging Italian social marketplace appreciated by important business associations for its innovative solutions in the charity 4.0 sector and for the important support provided to NPOs during their traditional function as well as against Covid-19 in Italy. Through a benchmark analysis, this work succeeds in highlighting the innovative solutions proposed by Charity Wall compared to the charity 4.0 systems on the market. More specifically, through the Charity Wall case study it is possible to demonstrate which aspects of Blockchain technology can be used to strengthen the philanthropic system by avoiding cases of fraud to the detriment of beneficiaries, receivers and donors as well as to create a closer network between the various philanthropic players to support charitable initiatives against the Covid-19.

## Introduction

The available literature provides countless insights that can allow us to investigate the evolutionary trends and the quality of the flows of donations to non-profit entities over time and under numerous perspectives. Authors such as James (2018); Mainardes et al. (2016); Bekkers and Wiepking (2011) provided important studies on donations flows by analyzing qualitative and quantitative as well as social aspects. Some authors instead pleaded an approach related to psychological principles, often due to the evidence of donations already made (Jacob et al. 2018) or the empathy of donors and the emotions aroused (Dickert et al. 2016). Furthermore, in such a context, the analysis of the motivations for giving has also come to lower and more practical principles; Wong and Ortmann (2016); Bekkers (2010); Karlan and List (2007); Vesterlund (2006) clarified some aspects related to material incentives and how price could influence the purpose of giving.

Nevertheless, in the context of the literature review, in our opinion some analyses emerged more than other. They clarify the close correlation between the legitimization factor (Diez 2018) the ability to create serious and efficient donation models (Wojciechowski 2009) as well as the reputation of non-profit organizations (Balsam and Harris 2014; Mews and Boenigk 2013; Maxson and Kuraishi 2011) and an increase in donations.

These studies – together with the numerous scandals related to the traditional charity processes that affected the way public perceives charity and increased the mistrust in charitable organizations – have provided an important starting point for delimiting the scope of our research.

In fact, in the last years and still today, charitable organisations must face several criticalities such as declining donations (NonProfit Quarterly 2019), skepticism over CEO pay (Charity Navigator 2016; Balsam and Harris 2014) and concerns about where donations end up (Charity Today 2020). This worrying situation is compounded by the dramatic criticalities brought about by the Covid-19 pandemic. In fact, while donations for the Covid emergency increased due to the public and private interventions, on the other side, the usual charitable flows were drastically affected.

There are numerous sources from the United States and the European Union that have dealt with this critical aspect.

As reported by CAF America: “Nearly all, 94.38% of the responding organizations, reported being negatively impacted by the coronavirus global pandemic. Over 70% of the respondents have seen a significant reduction in the contributions they receive and had to suspend programs involving travel or events” (CAF America 2020). According to the Association of Fundraising Professionals: “More than half of charitable organizations in the United States are expecting to raise less money in 2020 than they did in 2019, and an equal percentage believe the same will occur in 2021” (AFP 2020).

Unanimously with the AFP’s survey, during an online press conference the Italian Institute of Donation (IID) showed that on the first quarter of 2020 the 81% of NPO suffered a negative impact on fundraising and 40% of them reports a decline of more than 50% (Alvaro 2020).

The same situation is perceptible in U.K. as well, where charitable organisations have been widely affected by the Covid situation. The third sector has been impacted significantly with services being postponed and fundraising efforts scaled back. Small U.K. charities/nonprofits working overseas are facing innumerable challenges. A survey taken by Small International Development Charities Network highlighted that:

- 45% of charities working overseas will have to close this year without additional funding.
- 15% of charities working overseas will have to close within six months without additional funding.
- 77% of charities surveyed say that COVID-19 is already affecting their finances in this financial year.
- 59% said they had already or were currently accessing their reserves during the pandemic.
- 72% of charities surveyed said they had an increased demand for their services during the pandemic.
- 57% of charities surveyed said they have had to postpone programmes/projects during the pandemic.
- 66% of charities surveyed say they are responding to COVID-19 directly and a further 23% say they are responding in part.
- 64% of charities have found new ways to deliver services.
- Just 11% of charities say that they have been able to continue their work overseas as normal (Small International Development Charities Network 2020).

For this reason, as published on 20th of May, the U.K. Government has pledged £750 million to ensure VCSE (voluntary, community and social enterprise) can continue their vital work supporting the country, including £200 million for the Coronavirus Community Support Fund, along with an additional £150 million from dormant bank and building society accounts (Gov.UK 2020).

Therefore, while events, meetings and courses have been in the past an important element of the delivery strategy of charities, today innovation and increasing use of digital media and applications are helping to fill the gaps that the cessation of physical gatherings has left. At the same time, they can enable the charities to operate with reduced costs.

In fact, due to the self-isolation and lockdown volunteers are not able to collect on the streets; furthermore, the cancellation or postponement of mass events and individual or small group activities have brought community fundraising to a juddering halt. The level of need is certainly not the same for every charity but depends from their particular mix of income sources as well as from the specific impact of Covid-19 on their operating models; however, the numerous sources analysed clearly demonstrate the homogeneity of the negative impact.

In such a context, Blockchain technology is really strategic in order to improve the credibility of charities.

The Blockchain’s added value for philanthropy consists in transparency and accountability as well as in the clear evidence of the goals achieved (Wang et al. 2019). Furthermore, as we will demonstrate in this work, the Blockchain technology is able to:

- create a transparent relationship with donors and recipients as well as with other stakeholders;
- reduce the administrative costs through automation and through the smallest number of intermediaries;
- improve efficacy reaching the right people;
- acquire funds rapidly through crowdfunding;
- create a wider and complete synergy between the numerous players in the philanthropic system.

A further and specific aspect strictly related to the use of Blockchain for philanthropic purposes consists in cryptocurrencies’ advantages. In the last years hundreds of millions of dollars in cryptocurrencies have been donated, with notable donations including over $100 million to Fidelity Charitable, $29 million to Donors Choose, $4 million to The Ellen DeGeneres Wildlife Fund and many more (Youssef 2020).

According to Nonprofit Tech for Good’s 2019 “Global NGO Technology Report”, cryptocurrency donations represent 1%–5% of the payment methods used within the charity sector, with over 100% growth in some countries (Nonprofit Tech for Good 2019). Transactions in crypto currency, in fact, are immediate and without borders (Huberman et al. 2019). They are accepted even in countries where the banking system is less developed and eliminate the need for intermediaries. Furthermore, the transactions have no limitations of any kind and have a much lower cost than that of other traditional systems because factors such as the size of the transaction, the number of other transactions carried out simultaneously or the computational complexity of a smart contract are considered.

Crypto currencies are traceable (Easley et al. 2019; Yang et al. 2019). Donors can verify the different transactions and, therefore, verify where their funds have gone and if they have actually fulfilled the purpose for which they were collected. This allows donors to better choose where to allocate their future donations.

Traceability is an incentive for non-profit organizations to operate better, to reduce costs and to channel the greatest possible flow of money to beneficiaries.

As we will see, the accountability and cost reduction of charitable organizations is also increased by the specific and most used characteristic of Blockchain: the documentary and notarial function that allows greater efficiency in the exchange of documentation, an empowerment of entities, an increase in the security of documents that cannot be tampered with or deleted and of their staff and a reduction in printed paper.

Therefore, in order to spread the use of the Blockchain in the charitable sector as, also encouraging the development of charitable initiatives 4.0, it is necessary to explain the potential impact of this technology.

### The research framework

This work is structured in such a way as to demonstrate the impact of Blockchain technology on the development of the charity 4.0 sector. In order to better define the application context and the current challenges as well as the future ones, the authors considered it appropriate to analyse also the panorama in which Blockchain is already used and the criticalities that new initiatives encounter in the launch phase of their projects. Therefore, for a better understanding of the reader, the paper is structured in such a way as to:

1. define the current status of the Blockchain, identifying the main areas of application.
2. highlight the critical issues that are currently holding back the development of Blockchain initiatives and can therefore slow down the specific impact of this technology in the charity sector, through the literature analysis
3. define the specific application of the Blockchain in charity 4.0, highlighting its strengths compared to traditional systems.
4. demonstrate the important innovations that can enhance the charity 4.0 sector through the case study provided by Italian Charity Wall.
5. highlight the need to increase charity 4.0 initiatives due to the pandemic spread of Covid-19
6. define the important solutions provided by the charity 4.0 sector that can support the fight against Covid-19 through the Charity Wall case study.

## The current status of Blockchain

### The main areas of application

Over the last years, the promises of new technologies have revolutionized the social sphere thanks to the countless innovation incubators (Etzkowitz 2002; Smilor 1987) that are constantly experimenting new approaches in various fields (Hillemane et al. 2019; Lacity 2018; Al-Mubaraki and Busler 2017; Holiday 2014). This has led to hundreds of new organizations, businesses and initiatives focused on Blockchain (Richards 2019; Laplume 2018; Nofer et al. 2017; Nguyen 2016; Czepluch et al. 2015). To date, the main areas of application that the Blockchain has been able to permeate are best summarized as follows:

- Health;
- Financial Inclusion;
- Climate and Environment;
- Charity;
- Governance and Democracy;
- Digital Identity;
- Agriculture and Land Rights.

The study of these areas (Park et al. 2018; Galen et al. 2018; Lacity 2018; Elsden et al. 2018; Manski 2017; Casey and Wong 2017; Kokina et al. 2017; Park et al. 2015; Thomas and Schwartz 2014; Christopher 2011) has allowed to deepen several cases of application.

The rapid spread across the many areas is most likely due to the fact that organizations have discovered that the Blockchain is a reliable and accurate technological solution to address their daily challenges (Fischer 2018; Elsden et al. 2018; Gervais et al. 2016). According to several studies (Durnev et al. 2018; Manski 2017), the Blockchain has provided significant added value for achieving the social impact objectives of organizations. As evidenced by a study promoted by the Stanford University and the Center for Social Innovation (Galen et al. 2018), 66% believe that the Blockchain is an improvement over other methods, while 20% believe that it is necessary to solve their problems; only 14% think that it is a way to solve the problem, but that the other non-Blockchain solutions can be better.

The use of the Blockchain varies between the sectors due to the many intrinsic characteristics of them (Kamat and Seo 2019; Kamath 2018; Novo 2018; Kokina et al. 2017; Elgar et al. 2015; Nakamoto 2008), however, some general trends can be identified. As shown in Table 1, the most commonly found cases of use are “record and verification” and the use of the Blockchain for “payments and money transfers”. This indicates that this is a major problem between the different sectors. In the first case of use the Blockchain offers efficiency improvements (Qu et al. 2018; Deshpande et al. 2017a, b) and implementation (Manski and Manski 2018; Manski 2017) in the archiving (Miraz and Donald 2018) and simultaneous verification of records on multiple computers (Lee and Lee 2017) reducing the risk of data falsification (Zhu and Zhou 2016) and allowing individual users to access, track and share their data (Chen et al. 2017; Pilkington 2016).

**Table 1:** Potential critical consequences deriving from cases of fraud

Immediate consequencies: Revision of the transactions	Control over all invoices and accounting movements
	Check of bank accounts to estimate false payments
	Insurance claim
	Recourse to legal assistance for the recovery of stolen funds
Critical Operation Consequencies	Loss of credibility with donors
	Total revision of management systems
	Establishment of specific financial control committees
	Loss of collaborations with partner organizations

Source: authors’ elaboration

In the second field of application, i.e. payment and money transfer systems, Blockchain technology is optimal for building peer-to-peer networks (Gao et al. 2018; Peters and Panayi 2016) aimed at transferring money without relying on many intermediaries in the financial system (Dimitri 2019; Monaco 2015; Kiviat 2015; Dong et al. 2014). However, the study of the literature has shown that the launch of activities using the Blockchain has been more difficult than expected, whether for profit or non-profit initiatives.

### The main critical issues encountered during the launch and development of Blockchain-based initiatives

As is often the case in the launch phase of a new technology, the use of the Blockchain has its own critical issues that in some cases can turn into real challenges. These challenges relate to both internal and external aspects of organisations working with Blockchain technology, i.e. aspects that relate to the so-called sub-systems (Consorti and Venditti 2017) with which organisations come into contact on a daily basis (Rangone 2020) and which define the economic landscape of the various initiatives.

**Fig. 1:**
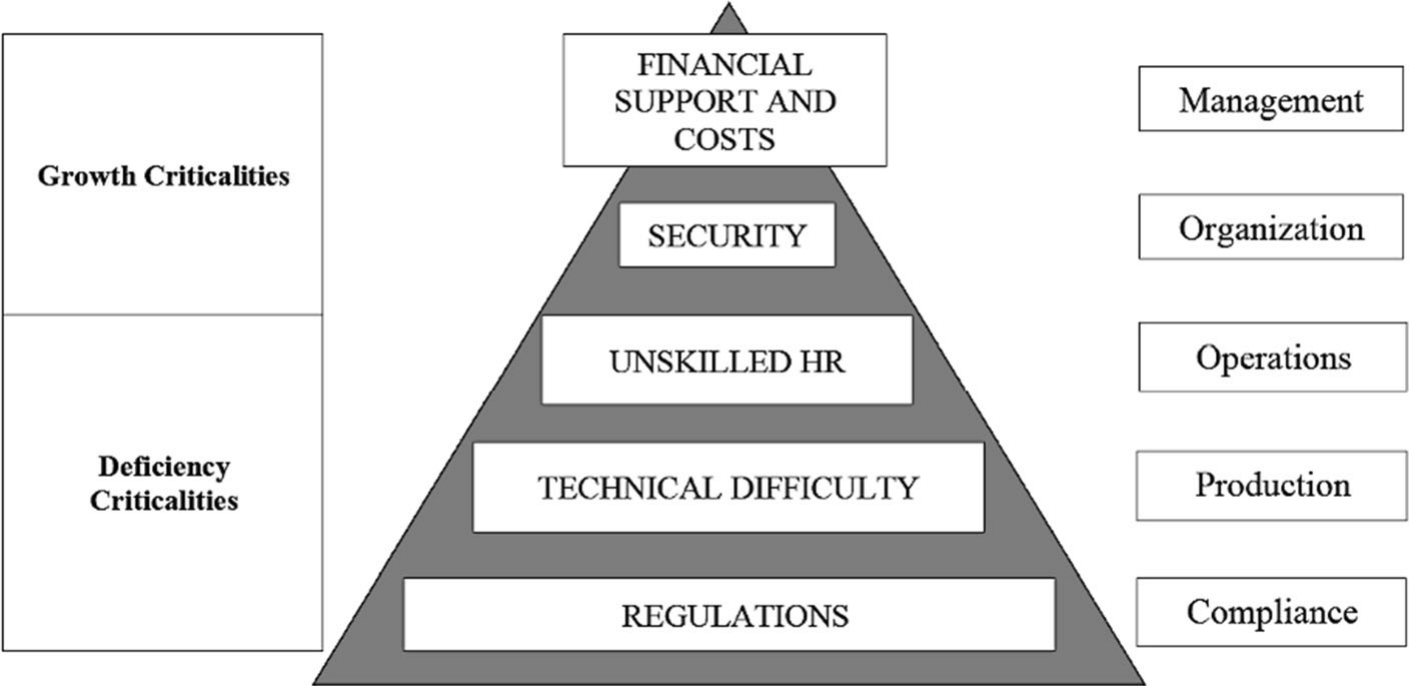
Main criticalities found in the use of the Blockchain by the study of the literature. Source: authors’ elaboration

The analysis of the literature has, in fact, made it possible to highlight countless problems (Fig. 1). The criticalities coming from the regulatory aspect are those most widely highlighted. The literature study and the clear evidences suggest that government regulations are not adapting quickly enough to properly incentivize (Smorgunov 2018) or facilitate growth (Deshpande et al. 2017a, b) of the Blockchain technology related initiatives, especially those one that can have a social impact. The improvement of policies and regulations in different areas of application of the Blockchain could catalyse more innovation (O’Dair and Owen 2019; Dong et al. 2014). A “hostile” regulatory environment or unclear regulations push organizations to operate in a “grey area”, or even in a lack of rules (Kim and Justl 2018).

Some have also reported that in their view regulators do not fully understand the technology and its potential impact (Deshpande et al. 2017a, b). Therefore, it is worth repeating, important policy measures are urgently needed to resolve this regulatory gap (Dierksmeier and Seele 2018) not only for the sake of a greater social impact (Johnson and Post 1996) but also to allow organizations working with Blockchain technology in compliance with a well-defined regulation (Werbach 2018). A further aspect that is highlighted among the critical points is related to the most typically productive area, namely the difficulty of finding technicians who are really prepared and who can contribute to the development of sophisticated projects.

This aspect, closely linked to the technical difficulties of the Blockchain, is far from slight. It depends on countless factors both internal and external, not only from the degree of technological acceptance by the companies of an area, the so-called *techno-corporate gap* (Rangone 2020), but also from the incentive to open innovation systems (Bijaoui 2015; Breunig et al. 2014; Fu 2012) and even more from the levels of education in the technical-informatics field (Zhuang et al. 2019; Zhao and Wang 2015). Further problems that define the top of the pyramid structure of critical issues in terms of impact are related to the costs of the initiatives and the level of financial support available on the market. The improvement of the organization or its ability to expand still depends too often on its ability to attract capital (Laplume 2018; Xia and Minshall 2013; Ahsan 2010).

Apart from important private equity firms (BarNir 2012), too many organisations are in a difficult situation to raise the capital needed for investment because the traditional banking system does not consider it appropriate to invest in their projects (Coleman and Robb 2012). Therefore, start-ups with an admirable initiative in the field of technology are forced to make up for the lack of capital by competing in public contests promoted by international organisations and with public development funds at stake.

## The use of Blockchain in charity sector

Donation and social impact platforms encourage social organisations (social enterprises, NGOs, charities) to manage projects in a transparent way. Blockchain solutions are designed to reduce financial and legal intermediaries and consequently also costs and time. In order to better understand the potential impact of Blockchain technology, it is essential to understand the system in which donations take place more generally. At the moment there is a great interest in the third sector because at a social level there is a great attention to humanitarian causes and people always respond strongly and reactively to difficult situations. The reasons for donating can be intrinsic:

- in order to contribute to a collective good;
- in order to respond to ethical and moral codes;
- in order to have a moral satisfaction (warm glow);

or extrinsic:

- economic incentives and material rewards;
- because they’re convincing (action of fundraisers).

However, it is right that everyone can be certain that their contribution has been successful. Too often it is possible to hear of scams and deceptions that not only leave the bitterness in the donor’s mouth, but in general damage the private charity sector of which today a modern state cannot do less. Today a “right to donation” should be guaranteed, as donation is a way to build the common good and to develop a community based on principles of reciprocity and unity. However, this right is not always respected. And it implies a progressive lack of confidence in the third sector with an increase in requests for transparency from donors and public administration and a consequent reduction in donations and public tenders. Let’s take a practical example. A substantial number of people have felt the desire to donate for the recent corona virus pandemic. Surely, they have wondered how their donations were used, whether for the stated purposes or for others. Thus, the purpose of the introduction of the Blockchain in the charity sector is to always be able to answer these questions and to avoid hearing from the media that money has not reached its destination, but has been used for something else.

### Technology against fraud cases at the expense of beneficiaries, receivers and donors

Too often it is possible to detect cases of fraud in the charity sector (AICPA and CPA Canada 2017; Greenlee et al. 2007) in which a coordinator of the charity association, a financial director or an operational supervisor in the implementation of humanitarian aid programmes misappropriated substantial funds. In this sense, as highlighted by the British Government in 2018 with the study of countless cases, the fraud at the expense of donors, beneficiaries and receivers can be numerous:

- the fraudster may be responsible for the payment of charity invoices without being an authorised signatory to the beneficiary institution’s bank account. Being able to access the credentials of the senior management team’s bank account to set up false beneficiaries, the fraudster can then transfer the funds to his bank account. The invoices are falsified and the fraudster uses the bank’s access data to authorise the false invoices (Gov.UK 2018);
- the organization’s management fraudster may improperly manage the distribution of donated amounts by manipulating the relevant accounting records (Gov.UK 2018);
- the operator supervising the charitable actions may divert sums fraudulently by means of deliberate overpayments for goods and documents which the beneficiary groups have not received. Often even false purchases have been charged to the charity program (Gov.UK 2018).

In these cases, the critical aspects following the fraudulent fact are numerous (Table 1) but above all they can affect the many players operating within the charity sector, encouraging distrust in donations to charity (Farber 2004). The Blockchain instead provides a clear system for tracking and certifying the use of funds in donations. This allows donors to constantly monitor, comment and verify the development of each specific social project. The aim is to ensure that, through a system of transparency and traceability, it can increase donor confidence in the third sector by encouraging donations in projects with a social impact that guarantee transparency and ensuring that donations actually reach those who need them.

## Empirical evidence on the application of the Blockchain in the charity sector

### Charity wall case study

Charity Wall (CW) is the most advanced and complete tool to trace and notarize the use of donations using the immutability and security of the Blockchain.

Charity Wall combines a Social Marketplace and an Automated Audit Solution for the charity sector.

Through the Blockchain, Charity Wall traces and notarizes the use of donations. Furthermore, it allows donating in total security as well as monitoring, commenting and constantly verifying the development of each specific social project. We can use the word Social to describe it both because it is oriented to serve the charity sector, and because it allows donors and receivers to create a story around the various social projects that any registered user can view and comment on.

Donations can be money and Charity Wall traces the contribution from the donor to the receiver and how it is spent. The donation can be also goods, in this case Charity Wall traces the fund-raising, the purchase, donation of goods and receipt by the non-profit organization and their use. CW aim is to ensure that, through a system of transparency and traceability, the trust of donors in the charity sector can increase, favoring donations to social impact projects that guarantee transparency and ensuring that donations actually reach those who need them.

Charity Wall wants to trace through Blockchain and make public all the documentation related to the activity of non-profit institutions and also to trace and convey in a complete way the donations flow among the various stakeholders.

CW applies the Blockchain to the fintech sector, to document notarization uses and to its utility token for the exchange and traceability of goods and services, giving the change to donors to monitor and comment each passage of the donation. Charity Wall is addressed both to donors that want to donate in total security and have the evidence how their money is used, and to non-profit organizations that want to transparently demonstrate the use of donations and with which they can also receive donations. Charity Wall add a layer of transparency to the total flow of donations. It can reduce the costs of charity by removing intermediaries, the papers of the stakeholder’s internal processes and improving digitalization and accountability. Furthermore, CW can reduce the costs by developing higher efficiency and faster procedures as well as enhanced donor base like crypto-donors. Although still a start-up, Charity Wall’s reality is therefore very young, it demonstrates a hacking growth (Ellis and Brown 2017; Ellis 2010) and the contribution it can make to the social sphere through the processes of donations has already attracted countless leading players. Assolombarda, for example, is the most important association of the entire Italian industrial system, called Confindustria. The partnership is under definition and it will allow Charity Wall to reach about 6300 companies of all sizes, national and international, producers of goods and services in all product sectors.

CW was selected to pitch in 2019 at Blockland Solutions in Cleveland (U.S.A.) and invited in December 2020 as speaker for the charity sector. CW was also selected as one of the most promising European Blockchain based startups by the European program Block.IS inside Horizon 2020. The core of Charity Wall is the marketplace where donors can meet virtuous NPOs and in which non-profit institutions can indicate the projects that need to be financed.

Through the marketplace donors and NPOs can show the projects they realized with the link to their project page, their pictures, videos, blogs and any kind of file related to the developed project. Donations, that can be either a money (30 crypto and 27 FIAT currencies) or goods ones, are supported by documents or files Blockchain uploads and certifications.

Charity Wall can trace in Blockchain all the passages of each donation, either money or goods one, thanks to its notarization system that allows to certify any kind of file in Blockchain. Charity Wall certification system allows knowing exactly who and when uploaded the file, giving the chance to share it and to make it downloadable, guaranteeing its immutability and its modification-proof across the time.

Charity Wall is the tool to exchange, share and download all the documents and files of each social project. Donations receivers must upload into the Charity Wall portal all the documents to trace the donation in Blockchain, and decide whether to make them public or to whom to show them and who allow to download them.

In case of money donations, they must upload all financial transaction documents to be traced in Blockchain. Donations receivers can also certify non-accounting documents to give greater clarity on how money is used and the results obtained.

Certified documents will be visible on project or receivers’ page, published in the Charity Wall marketplace (example: photos of airline tickets, or photos/videos of the assisted people thanking for the help, estimates that justify the choice of a supplier, etc.). Donations receivers can advertise their projects with photos, videos, and blogs and receive feedback, questions, and comments by donors. They will also receive a rating according to the proper use of Charity Wall by the Charity Wall algorithm. Charity Wall, by Ethereum and VeChain Blockchain, then, traces the money flow for and from the association and produces a complete document flow. Documents must match each other and - as they are public - they can be checked by users. In this way a false document or a modified one can be easily revealed.

### The potential of innovation promoted by charity wall

As also schematically identified in the benchmark prospectus provided by the figure n. 11, the strengths of Charity Wall from a technical point of view are numerous:

- Utility Token and marketplace;
- Blockchain transaction certificate;
- Widget;
- Money flow control both for income and expenditure;
- CharityPay (Charity Wall’s Payment system for donations);
- Donations from third parties’ websites;
- Double Blockchain System (Ethereum and VeChain);
- Donations in crypto and fiat;
- Certificate of advance donation;
- Web API;
- Documental check.

All these features make Charity Wall a complete tool to focus on the actual use of the donations and their traceability.

Charity Wall introduces a new and innovative way to donate, never seen before in the world of donations, through the marketplace and by its utility token CWC (Charity Wall Coin).

In the marketplace all stakeholders can donate and swap physical good and services with CWC.

Charity Wall wants to broaden the donation base of virtuous non-profit institutions. This is possible thanks to CharityPay which allows donations also in crypto currencies and not only in national currencies.

#### Utility token CW’s coin

The use of CW’s utility token is an important step for total transparency because it strengths the complete tracing of donations. It also allows to reward virtuous and transparent institutions that use Charity Wall, the exchange of goods and services through the marketplace between the various interested parties (for example, non-profit organizations, donor companies or other entities involved in the donation process, suppliers) and facilitates donations.

Creation of a VIP-191 token managed by a smart contract published on the Blockchain of VeChain allows to exchange asset usable through the marketplace created on the Charity Wall’s platform. It is a utility token that symbolizes a basic unit of measure and provides a unique value for the exchange of goods and services (working hours, goods) between the CW marketplace and stakeholders. Currently there are a multitude of tokens present on different Blockchain, and the vast majority of them were created and released to fund a project (ICO).

CW’s utility token does not aim to raise funds but to create an ecosystem in which the involved parts can obtain it according to and in proportion to their virtuosity and transparency, always respecting the rules defined by Charity Wall. The choice concerning the use of a VIP-191 token created and managed by a smart-contract published on VeChain’s Blockchain is dictated by the fact that a Permission less Blockchain is certainly much more suited to the principles of transparency, independence and correctness that Charity Wall aims at. Furthermore, Charity Wall believes that the VeChain community can give ample guarantees on the scalability of the network, on its continuous improvement and updating, nevertheless the use of a smart-contract allows to be able to implement particular and flexible logics to the process that one wants to create. Therefore, the various stakeholders can exchange utility tokens by transactions through the Charity Wall marketplace. The owners of CW’s coin can hold the tokens through their own private wallet and have their own private keys exclusively. They will be able to exchange them even outside the marketplace. In this regards, Charity Wall approach gives the following advantages:

- give non-profit institutions the opportunity to receive goods and services they need by providing other services in return;
- encourage transparency and adherence to the rules and values that Charity Wall describes in order to have CW’s coin available in exchange;
- use of CW’s coin also to purchase services or goods from companies that want to donate;
- also trace pro bono donations that are not following a normal flow of money.

In terms of technology, each CW’s coin is issued by Charity Wall through a smart-contract on VeChain’s Blockchain (VIP-191). The technology on which CW’s coin is developed is divided into the following layers:

1. The first layer consists of VeChain’s Blockchain for its characteristics of:

- to be a permission less Blockchain;
- its diffusion and security;
- its scalability and the continuous development that the developer community realizes;
- the feature of having a complete turing programming language for smart contract implementation.

2. The second layer consists of the smart contract developed by Charity Wall for the release and management of the CW’s coin in the form of VIP-191 Token which will allow:

- token creation and destruction;
- trace transactions through VeChain’s Blockchain and manage the exchanges of this asset;
- users to exchange tokens through the marketplace, store them on their personal wallets by holding private keys in order to allow free exchange.

3. The marketplace developed on the Charity Wall platform allows NPOs to exchange the token for performance or goods among them or with corporate, donors, professional, foundations, and anyone involved in the circuit.

In order to comprehend better the steps of the CW’s coin release and use process, it is possible to resume how it follows:

1. Charity Wall create and deposit tokens on its VeChain Wallet.
2. Charity Wall gradually release the CW’s coin to the NPOs registered on the Charity Wall portal, that will follow the transparency rules and according to their reputation based on the feedback received from the donors.
3. The NPOs will use the CW’s coin directly from their Wallet through their private keys by purchasing and exchanging services and assets from Donors, Corporate, other NPOs and any stakeholder.
4. Anyone will be able to use the CW’s coin through their private keys even outside the Charity Wall Marketplace.

#### Blockchain transaction certificate

**Fig. 2:**
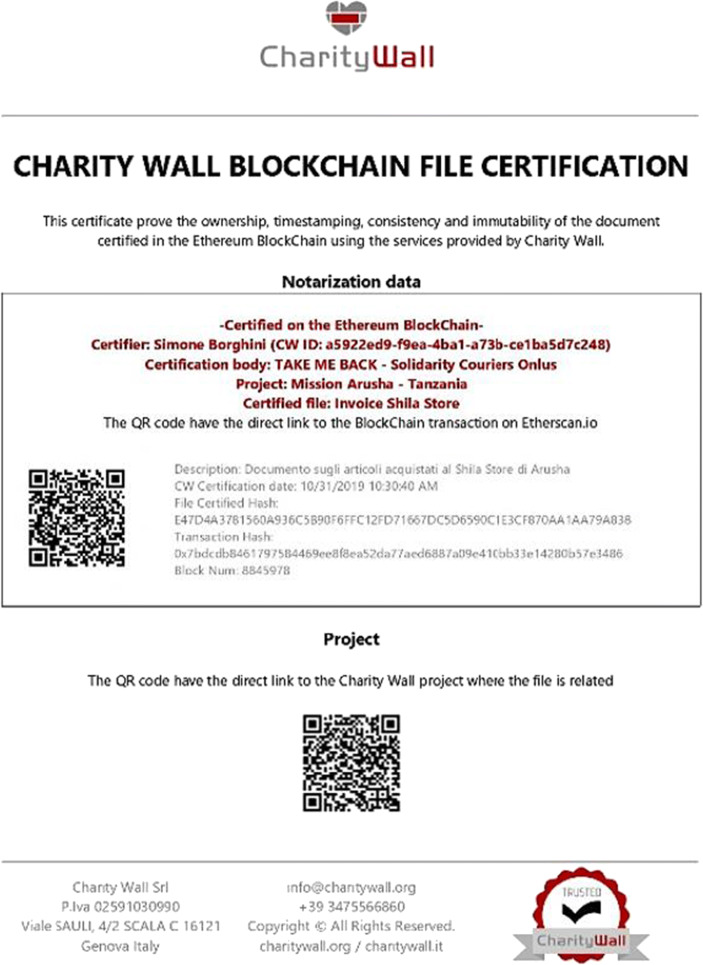
Blockchain transaction certificate, Source: Charity Wall

For each document certified in Blockchain through the CW system, Charity Wall issues a certificate. It is downloadable directly from the site and reports all the useful information relating to the transaction and the project. The certificate contains two QR Codes (Fig. 2).

The first leads directly to Etherscan and/or VeChain website (according to the used Blockchain), or the system made available to verify the transactions that took place. On Etherscan and/or VeChain website it will be possible to check the information on the certificate. The second QR takes you directly to the Charity Wall website to the project to which the certified document is linked, allowing you to download and view all related information.

#### Widget

The widget allows to view the following image on a website, showing visitors that the association uses the Charity Wall, giving Blockchain certified transparency of its activities. The widget refers to the information uploaded to the Charity Wall.

#### Donations in crypto and FIAT

Another important strong point ready to use is the donation in FIAT coins and currencies through crypto. It aims to trace how much the non-profit institution will collect for that specific project and how the institution spends the money.

#### Certificate of advance donation

It is also possible to track and certify in Blockchain all donations made outside the Charity Wall portal.

CW has created a feature that allows to certify the information relating to the donation in Blockchain such as who donated, who received, donation methods (bank transfer, credit card etc. etc.) and the amount of the donation. Therefore, it’s also possible to certify a document that can attest and report the donation made.

#### Web API

Charity Wall has introduced Web APIs, or web services, to allow third-party systems to communicate directly with Blockchain without changing their internal processes, but simply by calling the features made available.

Third-party systems can interact with the Charity Wall services both to update information and to certify documentation and donations in Blockchain.

### Documental check

**Fig. 3:**
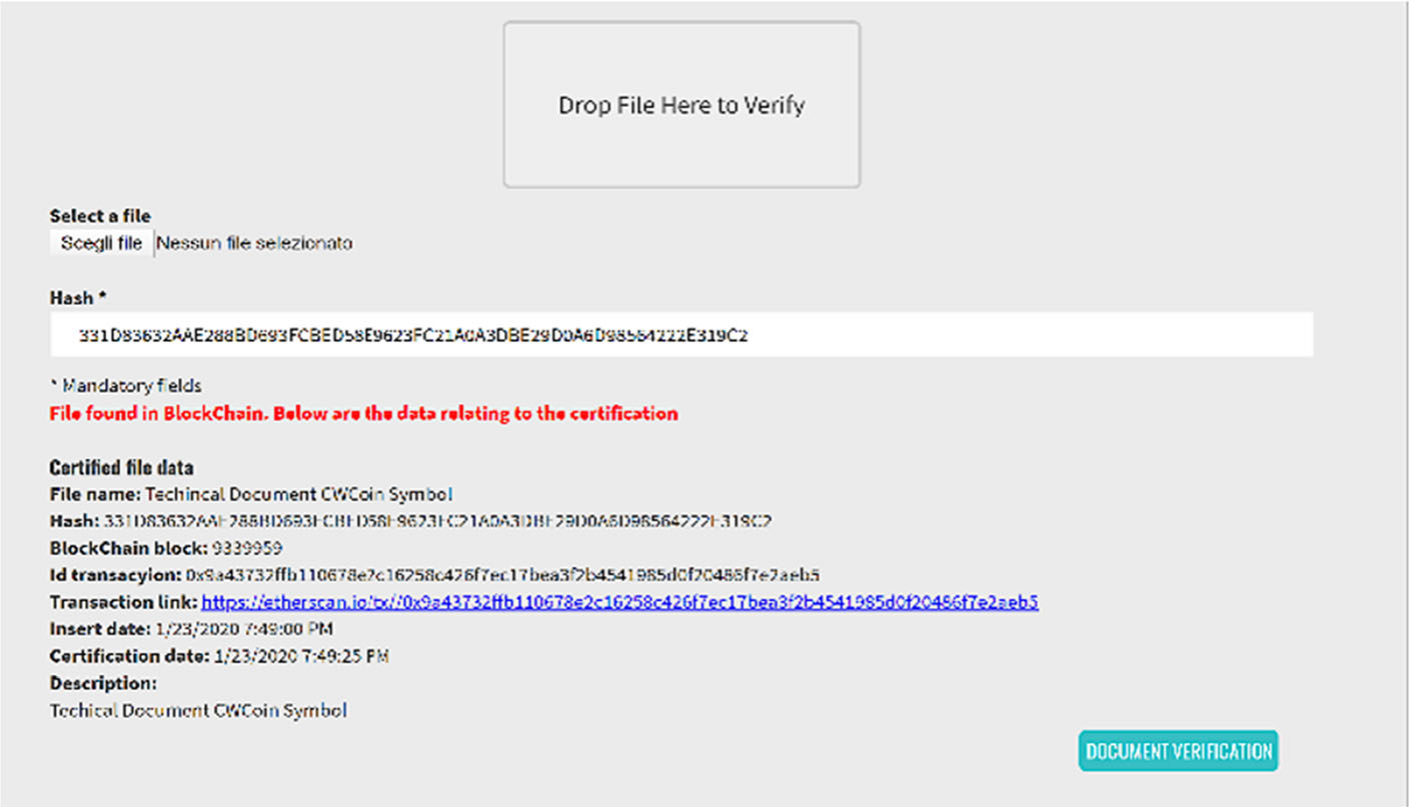
Documental check, Source: Charity Wall

CW makes it possible to verify that a document has been certified in Blockchain with a simple drag and drop (Fig. 3).

#### CharityPay

CharityPay is the Charity Wall’s payment system that can be used also on client’s websites or webportals to receive donations. Money goes to the e-wallet of the project opened in Charity Wall and is added to the amount of donations received from Charity Wall’s portal for the specific project. Money will, then, follow all the flow and steps of traceability by Charity Wall.

#### Double blockchain system

In order to guarantee a reduction in transaction costs, and not to burden the costs of associations, the Charity Wall solution is available both on Ethereum and VeChain Blockchains. This makes Charity Wall the first Blockchain company in the world to provide this choice, with a huge technological and economic impact.

#### Marketplace evolution

The marketplace is the heart of the new and innovative way to donate, never seen before in the world of donations, that Charity Wall has created using the BlockChain technology through the creation of its utility token CWC (Charity Wall Coin). In the marketplace all stakeholders can donate and swap physical good and services with CWC.

Once registered in Charity Wall, stakeholders can create, as in an e-commerce, products to donate to nonprofit institutions. Products identify physical goods, such as food, or services. Charity Wall creates a real charity market because the CWCs useful for the exchange are distributed for free according to the commercial methods and logics of Charity Wall (in example: payback, partnership, activities on the Charity Wall site, donations.

One of the token distribution channels is precisely the reward towards nonprofit institutions that prove to be particularly virtuous, following the transparency logic of Charity Wall.

In this way their ethical behavior will be rewarded because they will be able to acquire free goods or services donated by the various realities.

Furthermore, if donors increase the use of the portal the they get more CWC. In the commercial strategy of Charity Wall, it is also foreseen the stipulation of commercial agreements with partners to provide them with CWC as well as the development of CharityPay as a payment system which will include a payback logic in CWC. With the future evolution of the utility token to payment token, it will be also possible purchase CWCs.

### Further considerations about money donations and traceability

The document flow is supported by tracing the money transactions and the related documents.

When a donor (this can be single donors or large donors such as companies, foundations, banking foundations, philanthropic families) wants to finance a project or a non-profit institution the donation is sent to a specially created digital wallet. With the recent UpHold and Stripe partnership, fintech service provider, Charity Wall is able to accept donations in 30 cryptocurrencies and 27 national FIAT currencies.

**Fig. 4:**
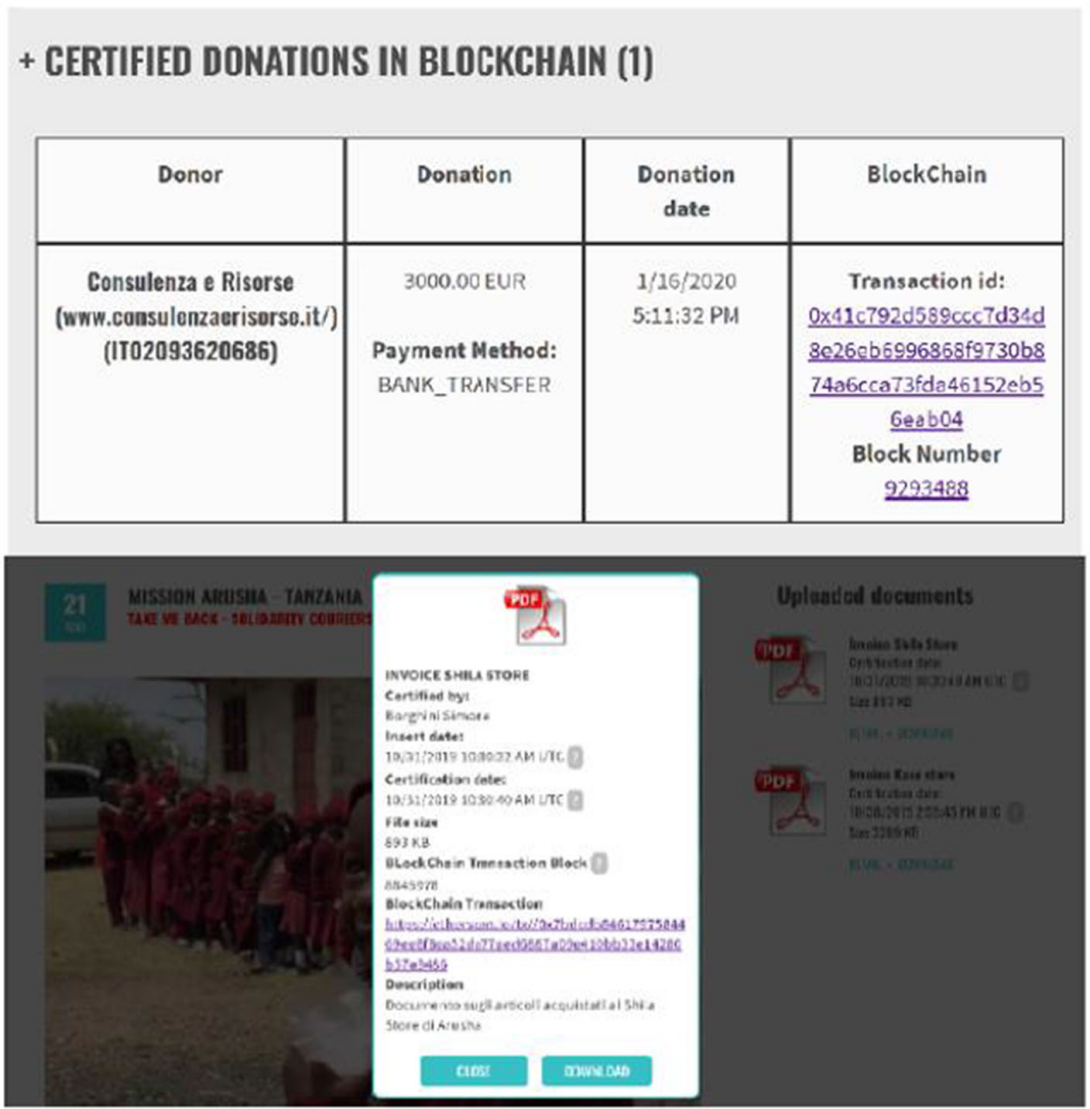
Donation certification and document certification. Source: Charity Wall

Each step is traced and certified in Blockchain, visible to stakeholders in real time (Fig. 4).

The e-wallet funds can arrive to the NPO or directly to suppliers or beneficiaries. Money receivers must upload into the Charity Wall portal all financial transaction documents to be traced in Blockchain. Furthermore, they can also certify non-accounting documents to give greater clarity on how money is used and the results obtained.

Certified documents will be visible on project or NPO page, published in the Charity Wall marketplace (example: photos of airline tickets, or photos/videos of the assisted people thanking for the help, estimates that justify the choice of a supplier, etc.).

The NPOs can advertise their projects with photos, videos, and blogs and receive feedback, questions, and comments by donors. They will also receive a rating according to the proper use of Charity Wall by the Charity Wall algorithm.

Charity Wall, then, traces the money flow for and from the association and a complete document flow. Documents must match each other, and, as they are public, they can be checked by users. In this way a false document or a modified one can be easily revealed. In the second stage of Charity Wall’s development, thanks to the payment token, the money flow from NPOs to suppliers will give a further support to transparency and will add further information that can make more easily verifying the truthfulness of the documents. Through these operations, the payment will be completely traced and each transaction will be associated via smart contract showing the payment details and all the information supplied to identify the use of the funds in full. The Blockchain tracking will allow to completely report all the phases involved in the project, giving immediate clarity and completeness of the information needed to provide complete transparency. This type of approach will permit CW to handle crypto money without having the private keys of the users and without concentrating all the donations on it.

At this stage it is possible tracing goods donations as well.

In this case Charity Wall traces the fund-raising activity, the goods purchase, the goods donation and delivery, the receipt by the non-profit organization and their use.

Donors purchase the items from the supplier, uploading into the Charity Wall portal and, then, in Blockchain all the documents (such as orders requests) to show their donations. Suppliers will upload the order received, the invoices, and all the documents related to the order. Also for the goods donations, NPOs can certify any kind of document and file to give greater clarity on how money is used and the results obtained. Goods receiver will upload the delivery note and any kind of file to show how they use the received material.

Certified documents will be visible on project or NPO page, published in the Charity Wall marketplace. The NPOs can advertise their projects with photos, videos, and blogs and receive feedback, questions, and comments by donors.

The Blockchain tracking will allow to completely report all the phases involved in the project, giving immediate clarity and completeness of the information needed to provide complete transparency.

## Charity 4.0 and COVID-19

As claimed by Ray Youssef (co-founder and CEO of Paxful) the most recent real-life use case for cryptocurrencies and charitable donations came in early January when the COVID-19 pandemic saw nations and communities facing shortages of personal protective equipment and medical support and having an overall need for immediate assistance (Youssef 2020). This opinion is also shared by Changpeng Zhao, founder and CEO of Binance: “The crypto community is a growing force and we have an opportunity to strengthen this through philanthropy. We encourage the community to take part in this initiative as we unite against COVID-19, and together, we’ll drive impact” (Binance Charity Foundation 2020). In fact, all over the world, doctors, nurses and medical resources are strained as the fight against the coronavirus continues. In response, many charitable organizations and philanthropists from various industries have come forward to offer their support.

In this regard, in the last months foundations and companies such as Stellar Foundation, Binance Charity Foundation and BitMEX undertook considerable fundraising campaigns against COVID-19.

By way of example, the Stellar Development Foundation launched a program to match donations given in Stellar Lumens (XLM), which was powered by Stellar-based Lumenthropy, a fundraiser that supports charitable organizations. Additionally, the Giving Block announced the start of the #CryptoCOVID19 alliance (Asia Times Financial 2020).

Binance Charity Foundation, with its campaign “crypto against covid”, raised 398.5646 BTC (the equivalent of 4,339,049.78 USD) from 178 donations, 24 Individual Crypto Recipients and 37 End-beneficiaries (Binance Charity Foundation 2020).

The Italian Red Cross, Municipal Committee 2–3 of Rome, opened in July a new fundraising campaign in BTC named “Bitcoin Saves Lives” to finance the purchase of essential products in order to continue to operate throughout the territory with more efficient means and tools. The President of the Italian Red Cross Municipal Committee 2–3 of Rome, the lawyer Francesco Pastorello, declared: “For our Committee this is a new approach to fundraising driven by the intention of bringing our supporters and the communities of people we assist as close as possible. With Bitcoin donations, donors will be able to directly check the impact of their contribution on our activities. We are sure that the generosity of Bitcoin holders will be of great help in the activities of the coming months” (CoinTelegraph 2020).

These are just some of the initiatives in the world that demonstrate the use of Blockchain technology in favor of initiatives against Covid-19. In the continuation of the work we will provide a more detailed analysis thanks to the Charity Wall case study.

### Charity wall and the fundraising campaign #DonaChiaro

In 2020, the Covid-19 emergency generates a pressing demand for protective material for doctors, healthcare professionals and patients.

Stocks are never enough and Italian hospitals are in a situation of extreme difficulty. Doctors and health personnel heroically work in conditions of urgency, extreme stress and the lack of protection systems subject them to the risk of infection. To supply the material to hospitals, it is necessary to purchase it and, therefore, to have funds available. Charity Wall wants to concretely help to fight the emergency and launches the first fundraising campaign based on the Blockchain technology. Charity Wall uses the Blockchain by tracking and certifying the entire flow of the donation, from its payment to its use. Furthermore, it allows donors to donate in total safety and to constantly monitor, comment and verify the development of each specific social project, through its storytelling, its documentation and related news updated by the proposing body and the actors involved. Charity Wall uses Ethereum’s permission less Blockchain, estimated to date at around 8000 “blocks”, as it is public, therefore not censurable and controllable by a single person, and for its characteristics of transparency and security. Through the Blockchain, Charity Wall allows the donor to verify all the steps of his donation as well as the use of the funds raised and any documentation, with the certainty that the information entered has never been altered by anyone. Furthermore, following the certification of a digital document, issues a certificate of authenticity with all the details to trace it, the association that issued it, the project to which it belongs and the detail of its registration on Blockchain. Charity Wall reduces the costs of charity by reducing the intermediaries, the documents of the internal processes of the interested parties, improving their digitization and accountability, allowing them to develop greater efficiency and faster procedures. In fact, the donation will be used to purchase material that will be donated directly to the hospital, reducing the costs and times of intermediaries or other associations.

On Charity Wall the path of the donated money is therefore traced and verifiable.

These two words constitute the strength of the #donachiaro campaign dedicated to the purchase of material for Italian hospitals. Donors, through the Charity Wall portal and thanks to the recent partnership with UpHold, can donate in 30 different cryptocurrencies or in 27 national currencies (FIAT).

The funds will go to the association’s account which then decides to unlock them to the supplier(s) of the material which will then be delivered to the hospital.

**Fig. 5:**
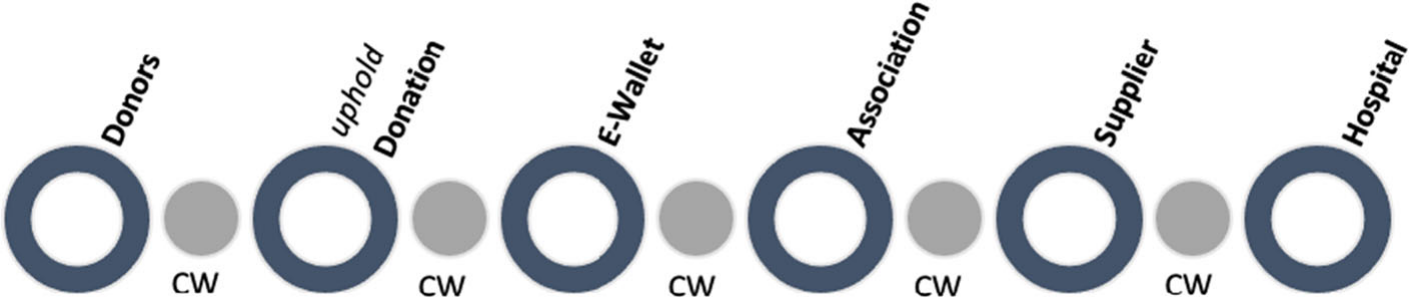
Donation flow #donachiaro in support of health facilities against Covid-19. Source: authors’ elaboration

The entire flow is certified in Blockchain and visible on the Charity Wall portal and, via widgets, also on the association’s portal (Fig. 5).

Charity Wall becomes the means and tool for uploading and sharing documents and files: the campaign promoting association can certify all the documents relating to the project in Blockchain and decide whether to make them public or to whom to show them and to whom to download. An example would be the receipt of the donation which is uploaded to the Blockchain and downloaded only by the relative donor. Similarly, the supplier can upload the invoice, delivery note and other documents that can be viewed and downloaded by the association or by all donors.

### A further tool in support of fundraising campaigns against Covid-19: Charity pay

During the validation process of the Charity Wall project, by carrying out surveys with existing customers and receiving feedback from possible future customers and from Block.Is mentors, the need emerged to evolve the package of CW solutions with something more versatile.

The need is to support all those realities that want:

to give the opportunity to donate and report on the use of donations directly from their website;

to raise funds also from third party sites;

to support those organizations that do not have a proprietary website;

to support those organizations that do not have the technological skills to integrate the Charity Wall Web API.

This is the reason why Charity Pay was created. It is not just a payment method but is a real system to create a wide synergy between the various players in the world of charity.

#### Charity pay configuration

**Fig. 6:**
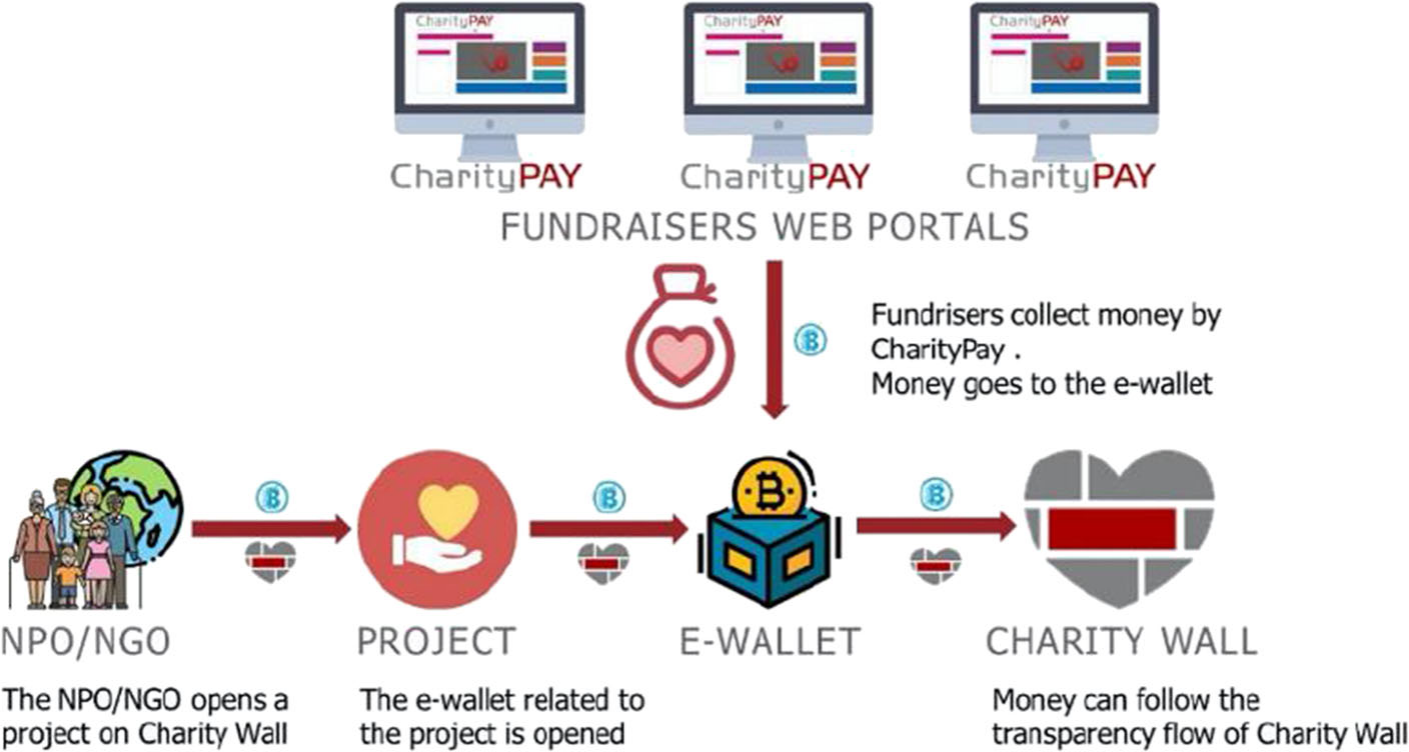
Charity pay process. Source: Charity Wall

Charity Pay is Charity Wall’s payment tool that can be used also on client’s websites or web-portals to receive donations (Fig. 6). Once the nonprofit organization opens the project in Charity Wall, it can collect money from Charity Wall’s website, but also from any website (for example: its website, partners’ websites, institutions or blogs or companies that want to spouse the cause).

Money goes to the e-wallet of the project opened in Charity Wall and is added to the amount of donations received from CW’s portal for the specific project, regardless of where they were collected.

Money and all donation processes are tracked in blockchain, by using the services and logic of Charity Wall as well as following all the flow and steps of traceability by Charity Wall.

CharityPay accepts:

- 27 National Currencies
- 30 Cryptocurrencies and Utility Tokens
- 6 Stablecoins
- 4 Metals
- Bank transfers
- Credit Cards

With CharityPay, then, the nonprofit institutions can enlarge received donations, thanks to:

- the possibility to receive donations for a specific social project from several websites, included Charity Wall’s one;
- the transparency given about the funds received and how they are spent;
- the donations in cryptocurrencies;

Through the back office of the Charity Wall portal it is possible to create a fully customizable HTML widget that can be easily inserted into any website that can be built custom or with wordpress solutions.

**Fig. 7:**
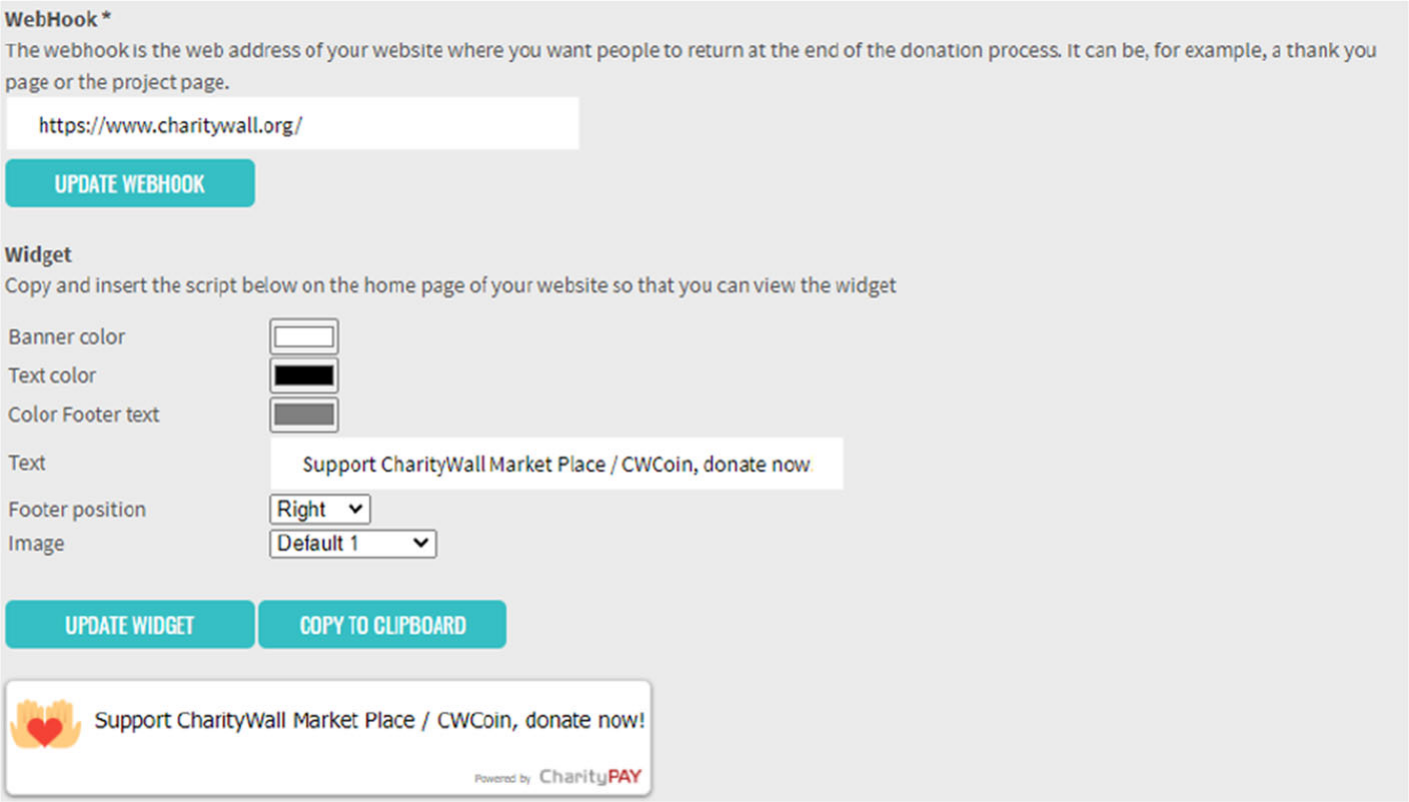
The widget. source: charity wall

This widget is easily customizable in all its parts (Fig. 7). It is possible to insert the “webhook” or a link pointing for example to a “thank you page” to which the donor will be redirected at the end of the donation process.

#### How the multi payment gateway works

The donation process has been structured to be multi payment gateway.

**Fig. 8:**
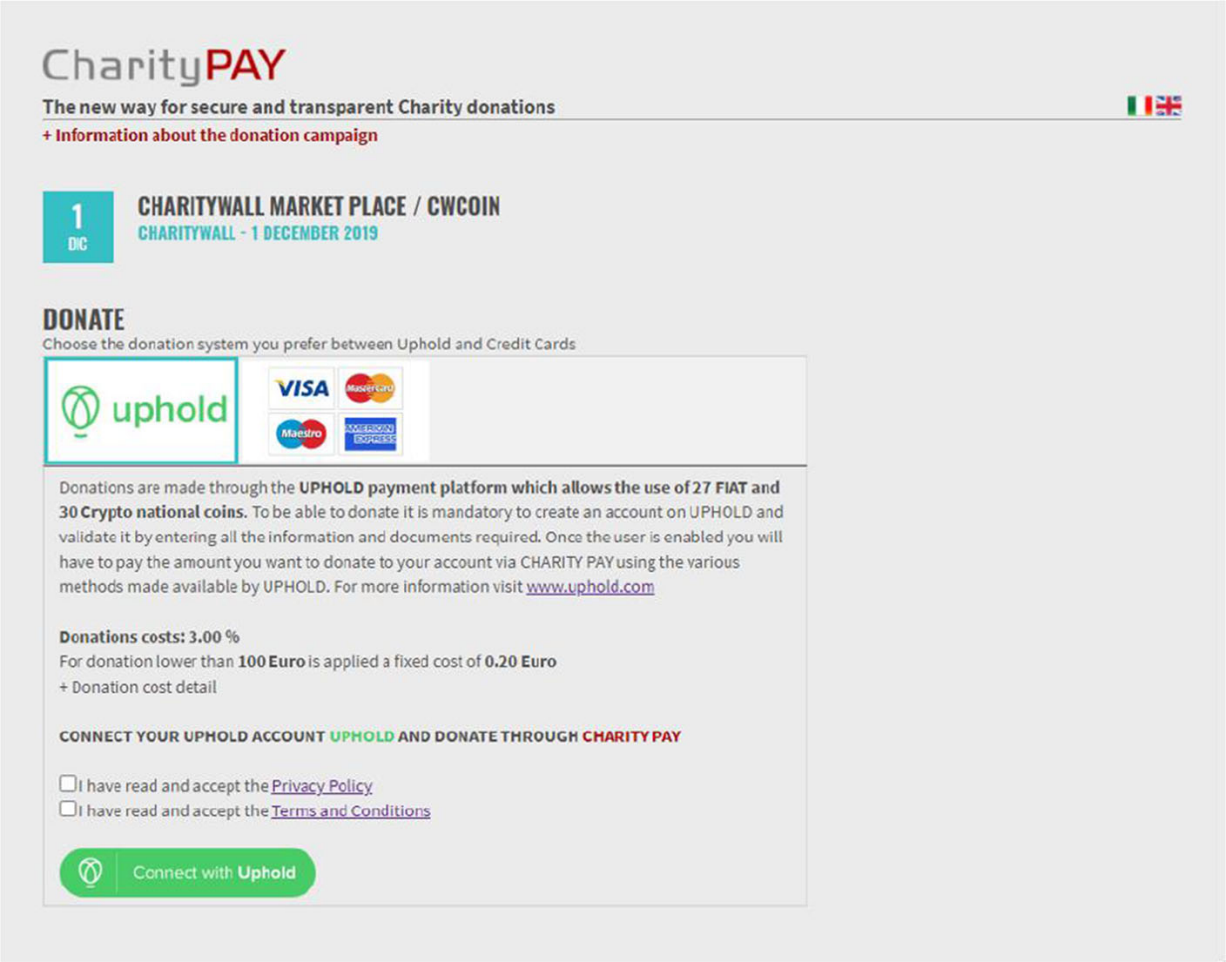
Donation process using Uphold. Source: Charity Wall

To date, the payment systems of “Uphold” and “Stripe” have been integrated (Fig. 8).

- “Uphold” is a solution mainly aimed at fundraisers and large donors such as companies, foundations or wealthy philanthropic families. It is also useful for those who want to donate in crypto currencies. The donor must register with Uphold, create their uphold account and load the money into that account.

Uphold allows donors to load their e-wallet even with high amounts, by choosing your preferred currency, and then be able to transfer the amount they want to the e-wallet of the project opened by the nonprofit institution.

b) “Stripe” is a suitable solution for small donors. In fact, it allows to pay directly by credit card, even without registering for the service, and is therefore useful for donating even small amounts of money.

**Fig. 9:**
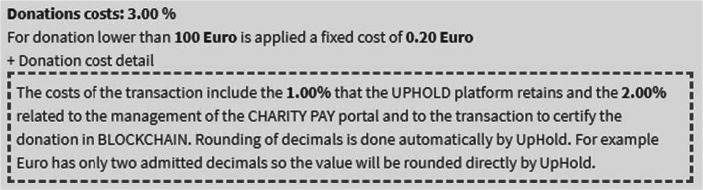
Evidence of the donation’s costs. Source: Charity Wall

Therefore, the donation process starts from the creation of the project on Charity Wall up to the sharing of the Charity Pay widget on multiple portals. This ability to share the widget allows donors to expand the collection to partner sites that can support the donation campaign with their network. Unlike the Charity Wall shared portal, shared between the various associations, the Charity Pay interface has been designed to avoid distractions for the donor, eliminating any possible reference to other projects or associations. Following the line of transparency of Charity Wall, expanding the cost box, the percentages that will be deducted from the donation with relative motivation are detailed (Fig. 9).

Therefore, Charity Pay is the ideal solution for all those associations that do not have the possibility to create their own website by providing a complete web space where it is now possible to:

- Donate via Uphold and credit card in crypto currencies and national currencies;
- See all notarized documents in BlockChain to report on the use of donations;
- Provide donors with a contact person for the association;
- Provide the association with a blog space in which to communicate with your donors, update them with news and project progress and where you can upload photos, videos and any other file useful for reporting on the use of donations.

By clicking “Detail” on a document, it is possible to view a whole series of information relating to the notarization in Blockchain and to the file in general.

**Fig. 10:**
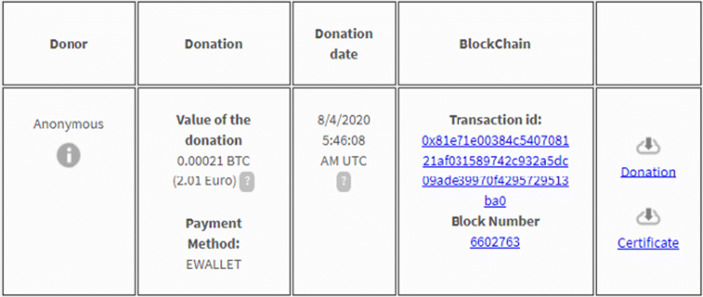
Notarization document. Source: Charity Wall

As previously mentioned, each donation is tracked in Blockchain. It is possible to download a certificate of the transaction in Blockchain and a summary document of the donation made (Fig. 10). It is possible to make anonymous donations or show the person’s name and surname.

If the user is not yet present in the Charity Wall systems, by donating a profile will be created automatically in order to allow the donor to manage their donations, receive all communications and participate in Charity Wall activities.

#### The development of charity pay

In the next future, Charity Pay will be integrated with CWCoin. Each payment corresponds to a payback in CW’s coin which can be donated to the NPOs/NGOs which can use them for the purchase of goods or services made available within the social marketplace.

Charity Pay will be a payment functionality directly applicable on purchase websites, e-commerce and sales platforms, and, through the app, physically in stores that want to use it. Each transaction between buyer and supplier corresponds to a payback in token to the customer to be used within the CW marketplace, while the supplier can decide whether to donate part of the proceeds to a social project among those present on the CW site. The donation transaction is traced in Blockchain.

## CW’s benefits to charity and the added value compared to current competitors

In order to comprehend the real added value Charity Wall provides to the social sphere, and in particular the charity sector, it is important to consider the concrete benefits obtained by the final users.

Schematically, it is possible to analyse the benefits both for donors and for non-profit institutions.

### Benefits for donors

Donors can donate in total transparency and monitor, comment and constantly verify the development of each specific social project. More specifically, they have the following benefits from the use of Charity Wall:

- Check the destination and use of donations at any time;
- Tell the story of the donation, obtaining a return of image and, thus, increasing customers for large donors such as donor companies or banking foundations;
- Have and provide certified documentation;
- Finding virtuous and transparent realities to which to donate;
- Greater simplicity to obtain CSR certifications.

### Benefits for non-profit institutions

NPOs have a tool to transparently demonstrate the use of donations and with which they can also receive donations. They have the following benefits from the use of Charity Wall:

- Give an image of transparency and reliability;
- Increase the received donations;
- Create a virtuous circle between donor and institution;
- Improve the accountability and the professionalization of the institution;
- Communicate what they have released and developed through certified data by the marketplace;
- Facilitate the work of professionals who work with the non-profit institution;
- Save time and resources for archiving documents.

More specifically, using Blockchain technology, Charity Wall is able to support NPOs during this delicate phase of recovery due to Covid-19 with regard to the following issues:

- Contributions reduced: by promoting easy, certified and traceable operations through Blockchain systems, CW can stimulate the donors’ trust.
- Travel restriction: Virtual operations allows to reduce and - in some cases completely avoid - the need for travels.
- Staffing disruption: through the Blockchain technology, CW allows to avoid the need for staff, volunteers and members, by reducing the number of intermediaries.
- Operations: CW’s Charity Pay system is able to sharing best practices, reduce possible negative impacts, mobilizing the network of partner as well as to create new partnerships with relief organizations.
- Increased costs: the operational costs reduction is the first measure adopted by charities during the Covid-19 pandemic. Through a completely digital philanthropic system, CW can drastically reduce the operational costs even maintaining the transparency of the operations.
- Supply chain broken: today, the most part of charities lack of access to the necessary infrastructure and technology to be able to benefit from the services offered online. Therefore, CW is able to reduce the supply chain of traditional philanthropic system.

**Fig. 11:**
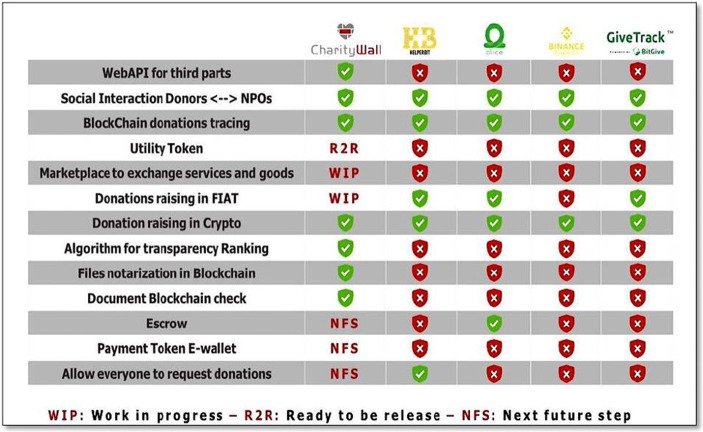
Benchmark of services and technology offered in the charity sector. Source: authors’ elaboration

There are charity players both in Europe and in U.S.A. However, the benchmark analysis (Fig. 11) shows that CW’s strengths consist in the marketplace, the web API for third parties, the token and the development of the escrow system that are combined with the possibility to upload and certify in Blockchain any kind of file. Today, some of these particularities are ready to be used or in progress to be released while the competitors seem to have no contemplated them from a qualitative and quantitative point of view.

## Conclusions

The Charity Wall case study highlighted several important aspects. First of all, we are experiencing a heavy and harmful reduction in donors’ confidence in philanthropy. This situation is mainly due to two reasons: the problems that the traditional charity system too often entails and the critical consequences of Covid-19. Nevertheless, the charity sector has untapped potential. It can be achieved by adopting Blockchain technology and stimulating the development of initiatives such as Charity Wall that can turn the donation system into a completely renewed process. As shown in the present work, this objective can regain trust in philanthropy by avoiding potential cases of fraud and misappropriation of charitable funds as well as economic, financial and social repercussions. The use of the Blockchain technology in the charity sector represents another dimension, an improvement that has nothing to do with what has been done so far. Money has always been collected and used in humanitarian funds, but today this process can be done better, more efficiently and in a totally transparent way. The Charity Wall case shows, in fact, that the technological potential for charity can be increased by offering new services, greater security and greater social impact, especially at a dramatic time marked by the Covid-19 pandemic. With initiatives like Charity Wall, the positive results for NPOs can be numerous: regaining the relationship with small and medium-sized donors, reducing operating costs, perfecting the entire supply chain and speeding up donation processes in favor of the final beneficiaries. Thus, there is an urgent need for a better awareness of the potential that Blockchain can bring from both the private and the public sectors. Only by gaining full awareness, this technology will be used exploiting its full potential, by stimulating new applications in the various fields of non-profit sector.

A second aspect has been contextually highlighted – albeit marginally – by this work and is closely related to the first. Studies on literature and the CW case suggest that Blockchain technology initiatives still encounter too many difficulties in the start-up and development phase due to community misunderstanding and unclear regulations.

Although this work focused on the application and added value Blockchain technology can bring to the charity sector through initiatives similar to Charity Wall, however, considering the additional evidence emerged during the analysis of the application context, we are committed to continuing studies on how to facilitate the development of initiatives similar to CW, hoping that this work will also stimulate other scholars to this end.
